# Optical Design and Lens Fabrication for Automotive Thermal Imaging Using Chalcogenide Glass

**DOI:** 10.3390/mi16080901

**Published:** 2025-07-31

**Authors:** Young-Soo Choi, Ji-Kwan Kim

**Affiliations:** School of Mechanical and Automotive Engineering, Gwangju University, Gwangju 61743, Republic of Korea; memschoi@gwangju.ac.kr

**Keywords:** automotive thermal imaging cameras, molded IR lens, chalcogenide glass, athermalization of optical system, compression molding

## Abstract

This paper is about the design and fabrication of infrared lenses, which are the core components of thermal imaging cameras to be mounted on vehicles. To produce an athermalized optical system, chalcogenide glass (As_40_Se_60_) with a lower thermo-optic coefficient (*dn/dT*) than germanium was adopted as a lens material, and each lens was designed so that defocus occurs in opposite directions depending on temperature. The designed lens was fabricated using a compression molding method, and the molded lenses showed less than 1.5 μm of form error (PV) using a mold iteration process. Through evaluations of MTF and thermal images obtained from the lens module, it was judged that this optical design process is obtainable.

## 1. Introduction

Far-infrared (8~12 μm wavelength) thermal imaging cameras have been mainly used for military purposes, as expensive core components (sensors and lenses) are used in their manufacturing. With the recent commercialization of less costly uncooled infrared sensors, thermal imaging cameras are being used in various commercial fields, such as the automotive, security, and medical care industries. In particular, various attempts have been made to use thermal imaging cameras as driving assistance systems and autonomous driving sensors by mounting them on vehicles [1,2,3]. Infrared lenses used in thermal imaging cameras have traditionally been manufactured by abrasive machining of crystalline materials such as germanium (Ge), but mass production is difficult and manufacturing costs are high, making infrared lenses difficult to apply in the automotive field. Recently, to solve this problem, a low-cost infrared lens was developed using chalcogenide glass. Chalcogenide glass is an amorphous material and can be used to mass produce lenses by applying the compression molding method, and is inexpensive compared to crystalline materials such as Ge [4,5]. Also, as chalcogenide glass has a very low thermo-optic coefficient (*dn/dT*) value (~20~90 × 10^−6^/K) compared to that of Ge (~450 × 10^−6^/K), the refractive index of the material does not change significantly even in high-temperature and low-temperature environments. This means that when an infrared lens is manufactured with chalcogenide glass having a low *dn/dT* value, it can be used without an additional device for athermalization, so it has properties suitable for thermal imaging cameras that are exposed to the external environment, such as automobiles [6,7]. As detailed studies on the optical design and fabrication of chalcogenide glass lenses for automotive applications are very rare, considerable research is needed. In this study, a fixed-focus infrared optical system considering athermalization for vehicle thermal imaging cameras was designed and fabricated using chalcogenide glass.

## 2. Experiments and Discussion

### 2.1. Optical Design

Table 1 shows the main design specifications of the optical system for vehicle thermal imaging cameras. The F/number is an important variable affecting not only the airy disk, but also the MRTD (minimum resolvable temperature difference). When the F/number is high because the noise is severe when acquiring images, it should be designed to be as low as possible [8]. The uncooled detector applied when designing the optical system had a 640 × 480 array and an effective pixel size of 10 μm, and the F/number of the optical system was 1.1 in consideration of the airy disk and the relationship with the MRTD.

As chalcogenide glass has a low thermo-optic coefficient value, infrared lenses made with this material are suitable for vehicle thermal imaging cameras, which easily deteriorate depending on low/high-temperature situations [7]. In this study, Schott’s IRG26 (As_40_Se_60_), which has a low *dn/dT* value, was used as a lens material; its properties are shown in Table 2. However, as chalcogenide glass has higher dispersion properties than Ge materials, it has the disadvantage that significant chromatic aberration occurs when it is used to make a lens. In general, refractive optical elements have positive dispersion properties, and because the refractive index is defined as a function of wavelength, chromatic aberration could be controlled by combining different materials. Diffractive aspherical lenses with diffractive optical elements (DOEs) applied to refractive optical elements have both the positive dispersion characteristics of refractive elements and the negative dispersion characteristics of diffractive elements. Thus, when diffractive aspherical lenses are used, chromatic aberration correction is possible without using an apochromatic lens in which three optical components are cemented together [9]. In this study, a diffractive aspherical lens was applied to correct both chromatic aberration and spherical aberration.

**Figure 1:**
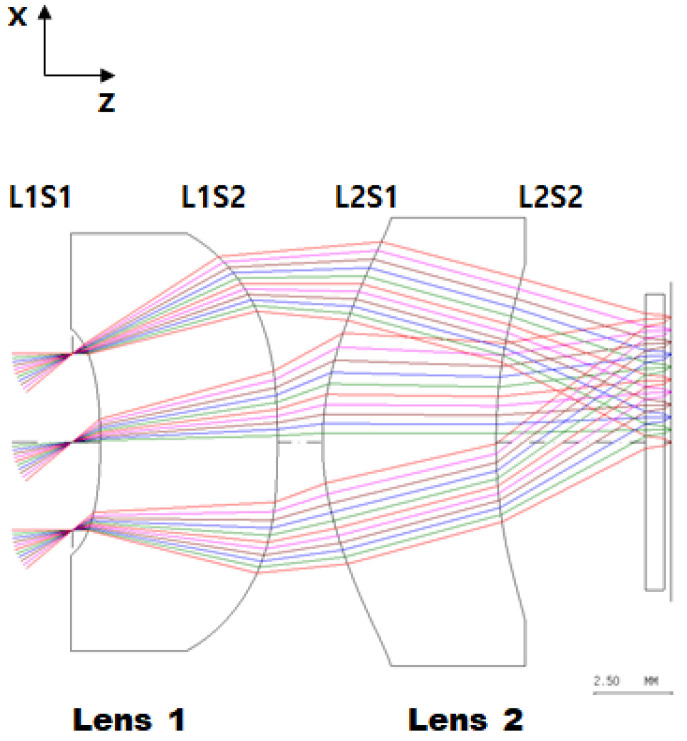
Layout of the optimized optical system.

Figure 1 is a schematic diagram of the optical system designed in this study and shows the ray tracing with the angle of incident rays. Table 3 shows the main design specifications of the designed optical system.

**Figure 2:**
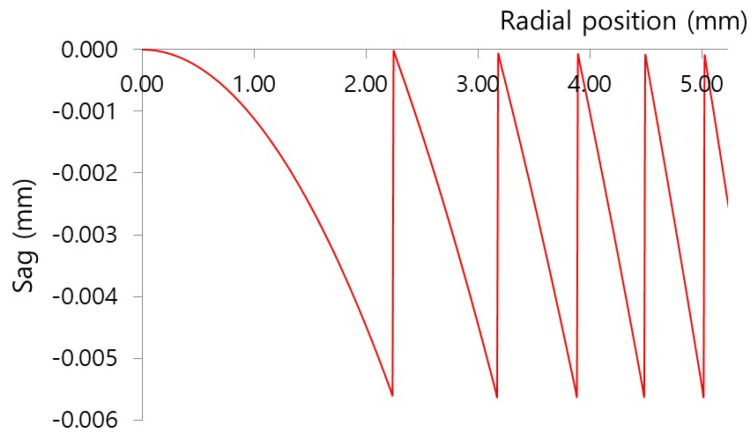
Surface sag of kinoform DOEs.

The designed optical system was composed of a double-sided aspherical lens and a diffractive aspherical lens made of chalcogenide glass material, and has a viewing angle of 61.6° × 45.5° (horizontal × vertical). Optical aberrations were minimized by employing an aspherical surface and a diffractive aspherical surface. The three surfaces (L1S1~L2S1) from the incident surface are aspherical, and the last surface (L2S2) is diffractive and aspherical. The aspherical surface corrects the spherical aberration of the optical system, and the diffractive surface corrects the chromatic aberration. The aspheric form is expressed as the sum of the cone and polynomial terms shown in Equation (1):

(1) $$z=\frac{C\cdot r^{2}}{1+\sqrt{1-(1+K)\cdot C^{2}\cdot r^{2}}}+\sum_{i=1}^{n}A_{i}\cdot r^{2i}$$

where $r=\sqrt{x^{2}+y^{2}}$ is the distance from the surface axis, *K* is a conic constant, *C* is calculated using the equation *C* = 1/*R*, *R* is the aspheric surface’s vertex radius, and *A_i_* is a constant for the aspheric form. The diffractive structure was designed in the form of a rotationally symmetric kinoform, and the phase profile is expressed as shown in Equation (2) [10]:

(2) $$\Phi \left(r\right)=m\frac{2\pi }{\lambda_{0}}\sum_{n=1}C_{n}r^{2n}$$

where *m* is the diffraction order, *λ*_0_ is the design wavelength, *C_n_* is the phase factor, and *r* is the radius of the diffraction ring. Equations (3) and (4) define the number and the depth of diffractive rings, respectively.

(3) $$N=\left(\frac{1}{\lambda_{0}}\right)\sum C_{n}r_{max}^{2n}=\frac{D^{2}}{8\lambda_{0}f_{0}}$$

(4) $$Depth=\frac{\lambda_{0}}{n_{0}-1}$$

where *D* is the lens diameter, *f*_0_ is the focal length of the kinoform DOEs surface, *λ*_0_ is the wavelength, and *n*_0_ is the refractive index at *λ*_0_. Equation (3) is an approximate formula valid under the assumption that DOEs exhibit a parabolic phase profile, as in the case of a diffractive lens. Table 4 and Figure 2 show the specifications and sag of the designed kinoform DOEs, respectively.

**Figure 3:**
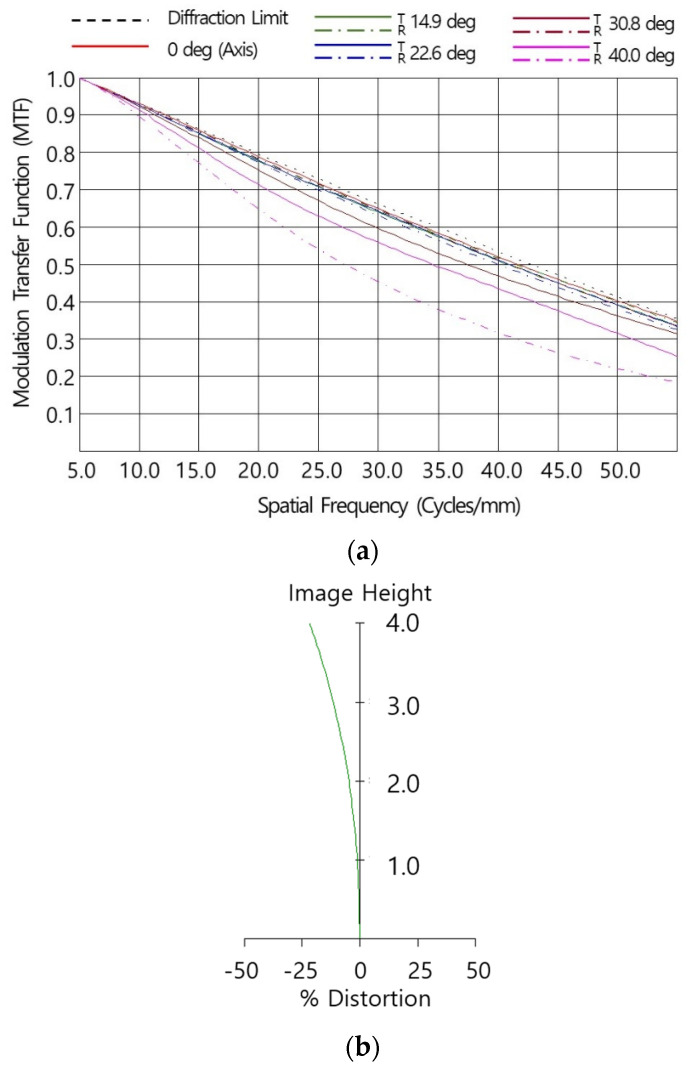
Optical performance of the optimized optical system: (**a**) optical distortion chart and (**b**) MTF chart at room temperature (20 °C).

The resolution of the designed optical system could be confirmed through its modulation transfer function (MTF) performance. The diffraction-limited MTF of an aberration-free optical system can be expressed by Equation (5):

(5) $$\begin{array}{ll} MTF(\xi /\xi_{cutoff})=\frac{2}{\pi }\left\{\cos^{-1}(\xi /\xi_{cutoff})-(\xi /\xi_{cutoff}){\left[1-{\left(\xi /\xi_{cutoff}\right)}^{2}\right]}^{\frac{1}{2}}\right\} & \mathrm{for}\;\xi \;<\;\xi_{\mathrm{cutoff}}\\ MTF(\xi /\xi_{cutoff})=0 & \mathrm{for}\;\xi \;>\;\xi_{\mathrm{cutoff}} \end{array}$$

where *ξ_cutoff_* $=\frac{1}{\lambda (fno.)}$. Figure 3a indicates the simulated MTF performance of the designed system at room temperature (20 °C) as a function of spatial frequency. The simulation was conducted using weighted wavelengths of 8 μm:10 μm:12 μm = 1:2:1. The MTF values of the designed optical system were lower than those of the diffraction-limited MTF due to optical aberrations, and it could be observed that as the incident angle increased, the MTF performance further decreased due to off-axis aberrations. At a 50 lp/mm Nyquist frequency the on-axis MTF was 0.35 and the horizontal angle (HFOV) MTF (Tan./Sag.) was 0.31/0.34, satisfying the requirements. Figure 3b shows a distortion chart of the optical system with the image height. Optical distortion is a deviation from ideal image geometry in which straight lines in the object appear curved in the image due to variations in magnification across the field of view. It had a distortion of −21.7% on the detector diagonal position, which satisfied the optical requirement of less than |25|% for a given detector (pixel size 10 μm, 640 × 480 array).

### 2.2. Athermalization Design of the Infrared Optical System

**Figure 4:**
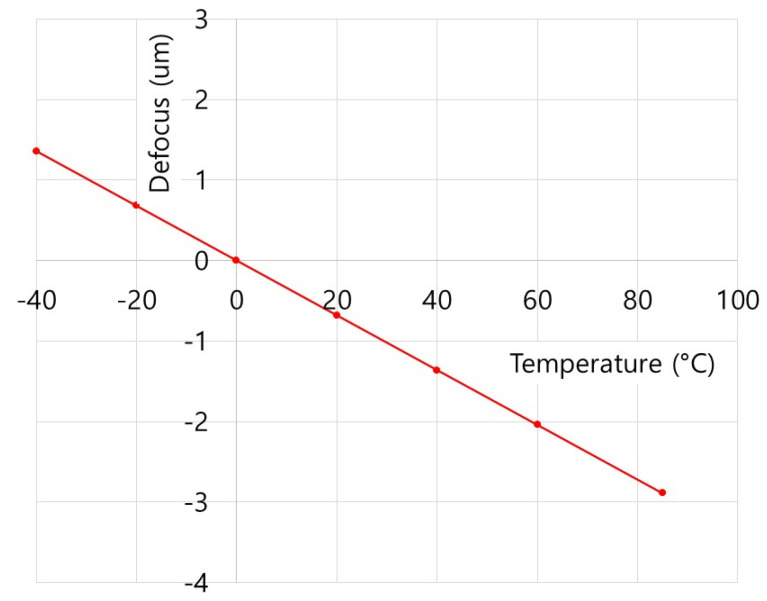
Thermal defocus of an optimized optical system.

**Figure 5:**
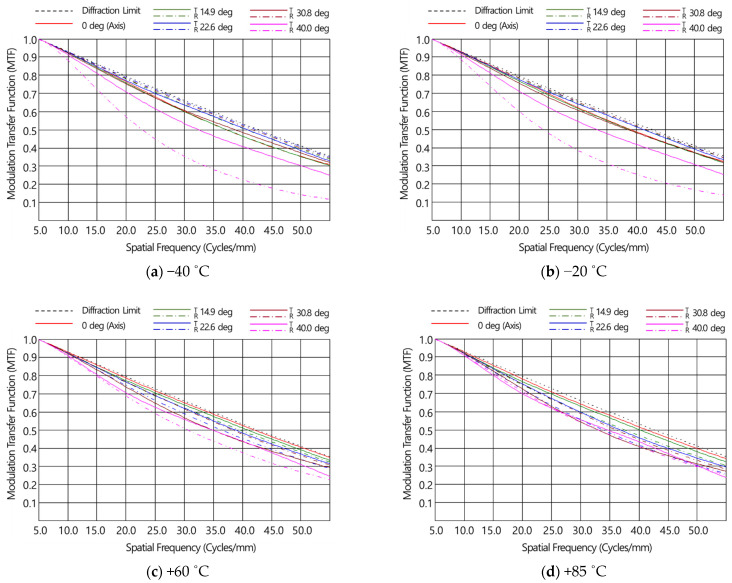
MTF charts in the temperature range of −40~+85 °C.

Infrared optical materials are useful for design because they have a very large refractive index compared to oxide glass, which is used in visible optics, but they have a problem in that the performance of the optical system changes sensitively as temperature changes. Although this performance change could be caused by the aberration characteristics of the optical system, in most cases it is induced by defocusing. The change in focal length is caused by changes in both the constituent materials’ thermal expansion coefficient and the lens material’s refractive index. Table 5 shows the value of materials used in this study. Assuming a thin lens, the change in the focal length of the optical system as the temperature varies is expressed as Equation (6) [8]:

(6) $$\frac{df_{r}}{dT}=-f_{r}[\frac{dn}{dT}\left(\frac{1}{n-1}\right)-\alpha ]$$

where *f_r_* is the focal length of the thin lens, *α* is the thermal expansion coefficient of the material, *n* is the refractive index of the lens material, and *dn/dT* is the thermo-optic coefficient, representing the change in the refractive index of the material as the temperature changes. Table 6 shows the change in focal length as temperature changed of the designed optical system in this study. As can be seen in Table 5, as the focal length changes in L1 and L2 as the temperature changed were in opposite directions, the defocus generated by temperature changes was compensated. It was confirmed that the defocus amount of the designed optical system in the range of the operating temperature (−40 °C to 85 °C) was from 1.36 μm to −2.89 μm, as shown in Figure 4. As the defocus amount was very small, the performance change in the system as the temperature changed was not expected to be significant. Figure 5 shows the MTF simulation performance of the designed optical system in the operating temperature range of −40 °C to 85 °C, and it was confirmed that the MTF performance was 0.2 or higher from the on-axis (0°) to the horizontal angle (HFOV) of 61.6° (half-view angle of 30.8 deg). The temperature-specific MTF charts result from applying both the thermal expansion of the material and the shift in the focal length of the lens under the corresponding temperature conditions.

### 2.3. Molding of Chalcogenide Glass Lens and Thermal Imaging Evaluation

**Figure 6:**
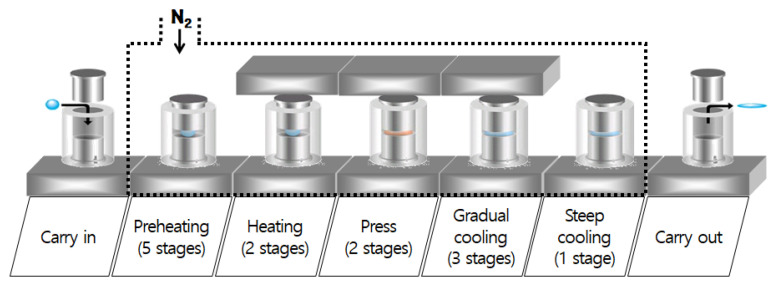
Schematic diagram of molding process in LMR-3300V2S.

**Figure 7:**
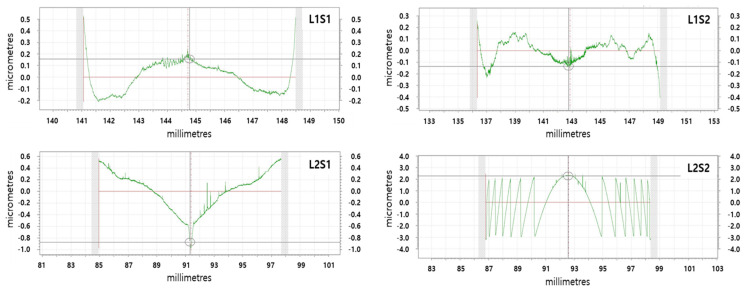
Form errors of the molded lens.

**Figure 8:**
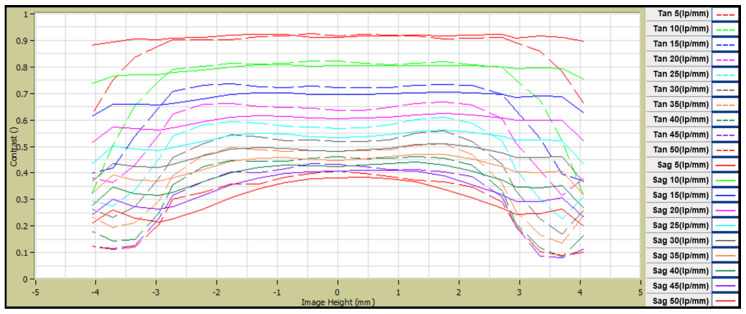
MTF chart of lens module at room temperature (+20 °C).

**Figure 9:**
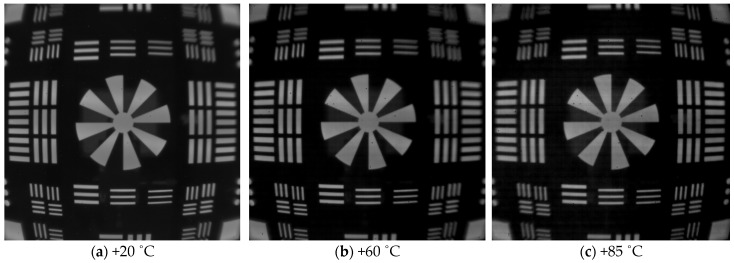
Thermal images of prepared lenses at temperatures of +20 °C, +60 °C, and +85 °C.

The lens core for molding chalcogenide glass was fabricated using a tungsten carbide (WC, R07, Nippon Tokushu Goukin Co., Toyohashi, Japan). The mold core’s surface was ground with aspheric and diffractive–aspheric forms and then polished to improve roughness. A diamond-like carbon (DLC) thin film of 150 nm thickness was coated to prevent glass adhesion on the mold core’s surface and to enhance the lifetime of the mold. We used ball-shaped preforms to mold the lens; it was composed of As_40_Se_60_ (IRG26, SCHOTT Co., Mainz, Germany), the thermal properties of which are shown in Table 2. Chalcogenide glass lenses were molded using a precision glass-molding machine (LMR-3300V2S, Daeho Technology Korea Co., Ulsan, Republic of Korea). The molding process of the LMR-3300V2S consists of five major steps: preheating, heating, pressing, gradual cooling, and steep cooling, as shown in Figure 6. At the preheating step, the mold assembly includes a preform that is heated from the bottom plate under no load. Next, this assembly is heated by both the top and bottom plates in the heating step. In the gradual cooling step, the heated glass preform is pressed with constant pressure in the pressing step, and the pressed lens is then slowly cooled to release the induced stress inside the lens. Finally, the molded lens is rapidly cooled using N2 gas and released from the mold. Table 7 summarizes the details of the process parameters and molding conditions used in this study, which were determined based on previous studies [9,11,12,13]. The molded lenses showed several microns of form error (more than 10 μm), which was higher than that of the lens core (about 0.5~1 μm); this was caused by thermal expansion of the mold at high temperatures and by glass deformation during the molding process. A form error of over 10 μm becomes detrimental to the lens for the LWIR imaging system in general, which requires less than about 2 μm as a PV (peak-to-valley) value [14]. Therefore, to mold high-precision lenses, it is desirable to design the core’s shape with a small amount of offset. This geometric offset is usually achieved by a mold iteration process, in which molding tests are carried out repeatedly with different core geometries until the lens meets the form error requirements [12,15]. Figure 7 depicts the form errors of the molded lenses, which were compensated for using the mold iteration process in this study. It could be seen that the form of the lens core was well transferred to the lens without defects in both the diffractive aspherical surface and the aspherical surface, and both Len1 and Lens2 showed a form error (PV) lower than 1.5 μm. The molded lens had an average transmittance of 65% in the range of 8–12 μm, and an anti-reflective coating was deposited on the lens surface to increase the average transmittance to 95%. The coated lens was assembled to prepare a lens module, and the MTF was measured at room temperature. The MTF of the lens module was measured using MTF equipment (ImageMaster IR, TRIOPTICS GmbH, Wedel, Germany), and the results are shown in Figure 8. At a 50 lp/mm Nyquist frequency, the on-axis MTF (Tan./Sag.) was 0.4/0.37, and the MTF (Tan./Sag.) of HFOV with an image height of 3.2 mm was about 0.24/0.15. The resolution of the lens prepared based on the MTF was slightly lower than the design value, which was judged to be due to fabrication tolerances such as lens tilt, lens decenter, housing tolerance, and so on. The athermalization performance of the prepared lens module was qualitatively evaluated by comparing the thermal images of a room-temperature lens and a high-temperature lens. When a lens cooled below zero, water droplets condensed on the lens surface, making image acquisition impossible. To obtain a thermal image, a room-temperature lens was first mounted on the thermal imaging camera (sensitive to wavelengths from 8 to 12 µm) to optimize the lens position, and then a thermal image was taken. Next, after keeping the corresponding lens in a high-temperature chamber (+60 ˚C and +85 ˚C) for 3 h, the lens was mounted at the same position optimized for the room-temperature lens to take a thermal image. Images taken using room- and high-temperature lenses are shown in Figure 9. Thermal images taken using the high-temperature lens appeared to be slightly deteriorated compared to the thermal image taken by the room-temperature lens, but there was no significant difference, so it was judged that the athermalization process adopted during the lens design is obtainable.

## 3. Conclusions

This paper reports the design and fabrication of infrared lenses using chalcogenide glass materials, to be mounted on vehicles. The optical system consists of two lenses, including three aspheric surfaces and one diffractive–aspheric surface, which were introduced to compensate for chromatic and spherical aberration. In addition, to fabricate the athermalized optical system, chalcogenide glass (As_40_Se_60_) with a lower thermo-optic coefficient (*dn/dT*) than germanium was adopted as a lens material, and each lens was designed so that defocus occurs in opposite directions depending on temperature. The designed lens was fabricated using a compression molding method capable of mass production and the mold iteration process was adopted to mold the precision lens with less than 1.5 μm of form error. The MTF of the lens module, which was an assembly of molded lenses, was measured to conform to the resolution performance. The MTF values were slightly lower than the design value, which was judged to be due to fabrication tolerances such as lens tilt, lens decenter, housing tolerance, and so on. The athermalization performance of the prepared lens module was qualitatively evaluated by comparing the thermal images of a room-temperature lens and a high-temperature lens. Through evaluations of MTF and thermal images obtained from the lens module, it was judged that the optical design process is obtainable. In the future, a study will be conducted to quantitatively evaluate the athermalization performance of these designed lenses.

## Figures and Tables

**Table 1:** Optical requirements for an optical system.

Parameters		Requirements
Detector		640 × 480, 10 μm
Spectral range		8.0~12.0 μm
F/number (fno.)		1.1
Field of view (Horizontal × Vertical)		61.6° × 45.5°
MTF @50 lp/mm	(Center)	≥0.3
	(HFOV)	≥0.25
Optical distortion		≤|25|%
Athermalization (−40~+85 °C)		MTF (@ 50 lp/mm; *On-axis*) ≥ 0.2

**Table 2:** Properties of chalcogenide glass (IRG26).

Term	Unit	Value
Transition temperature (T_g_)	°C	185
Thermal conductivity	W/m·K	0.24
Thermal expansion coefficient (@20 °C)	10^−6^/K	20.8
Thermal change (*dn/dT*) (@10.6 μm wavelength)	10^−6^/K	32.2
Softening temperature (T_s_)	°C	236

**Table 3:** Design data of the optimized optical system.

Scheme		Radius (mm)		Thickness (mm)		Material	
1. STO		Infinity		0.9		-	
2. L1		−15.1		5.7		IRG26	
3		−32.3		1.45		-	
4. L2		8.2		5.6		IRG26	
5		19.4		4.8		-	
6		Infinity		0.6		Si	
Surface No.	2		3		4		5
*K*	0.0000		0.0000		0.0000		1.1103
*A*_4_	−1.9503 × 10^−5^		1.9486 × 10^−4^		−3.1136 × 10^−4^		−1.8326 × 10^−5^
*A*_6_	−4.9027 × 10^−6^		−4.6883 × 10^−6^		−1.8813 × 10^−5^		−2.1507 × 10^−6^
*A*_8_	3.8746 × 10^−7^		−4.7384 × 10^−8^		2.3311 × 10^−6^		1.0116 × 10^−7^
*A*_10_	−4.8155 × 10^−0^		−1.2605 × 10^−7^		−2.5402 × 10^−7^		−2.7805 × 10^−9^
*A*_12_	1.6997 × 10^−9^		5.8932 × 10^−9^		1.2779 × 10^−8^		3.6328 × 10^−11^
*C*_1_	-		-		-		−0.9165 × 10^−3^
*C*_2_	-		-		-		0.2132 × 10^−5^

**Table 4:** Specifications of the diffractive aspherical surface.

Refractive Index [*n*_10μm_]	Clear Aperture	Depth	Number of Rings
2.7781	10.45 mm	5.63 μm	5.4

**Table 5:** Material properties.

Element	Material	Refractive Index [*n*_10μm_]	Abbe Number [*ν*_8–12μm_]	*α* [×10^−6^/K]	*dn/dT*_10μm_ [@−50~75 °C; ×10^−6^/K]
Lens	IRG26	2.7781	161	20.8	32.2
Housing	Aluminum (AL6061)	-	-	26.3	-

**Table 6:** Focus shifts as temperature changed.

Lens	Material	EFL(mm) @20 °C	(*dn*/*dT*)/(*n* − 1) [×10^−6^/K]	*df_r_/dT* [mm/K]
L1	IRG26	−18.60	18.1	−0.000050
L2	IRG26	5.96	18.1	0.000016

**Table 7:** Process parameters and molding conditions used in this study.

		Preheating	Heating	Pressing	Gradual Cooling
Temp. (°C)	Top plate	-	245	260	100
	Bottom plate	180	245	245	100
Pressure (MPa)		-	-	0.2	0.05
Unit-processing time (s)		300			

## Data Availability

The original contributions presented in this study are included in the article. Further inquiries can be directed to the corresponding author.
